# Food store owners’ and managers’ perspectives on the food environment: an exploratory mixed-methods study

**DOI:** 10.1186/1471-2458-14-1031

**Published:** 2014-10-03

**Authors:** Clarence C Gravlee, P Qasimah Boston, M Miaisha Mitchell, Alan F Schultz, Connie Betterley

**Affiliations:** Department of Anthropology, University of Florida, 1112 Turlington Hall, PO Box 117305, Gainesville, FL 32611-7305 USA; Department of Children & Families, Substance Abuse and Mental Health Program Office, 1317 Winewood Blvd, Bldg 6, Rm 256, Tallahassee, FL 32399 USA; Project FOOD Now, Inc, 635 E. College Avenue, Tallahassee, FL 32301 USA; Tallahassee Food Network, Inc, P.O. Box 365, Tallahassee, FL 32302 USA; Greater Frenchtown Revitalization Council, 812 Goodbread Lane, Tallahassee, FL 32303 USA; Department of Anthropology, Baylor University, One Bear Place #97173, Waco, TX 76798-7173 USA; Leon County Health Department, 2965 Municipal Way, Tallahassee, Florida 32304 USA

**Keywords:** Food stores, Food environment, Health inequalities, Formative research

## Abstract

**Background:**

Neighborhood characteristics such as poverty and racial composition are associated with inequalities in access to food stores and in the risk of obesity, but the pathways between food environments and health are not well understood. This article extends research on consumer food environments by examining the perspectives of food-store owners and managers.

**Methods:**

We conducted semistructured, open-ended interviews with managers and owners of 20 food stores in low-income, predominantly African American neighborhoods in Tallahassee, Florida (USA). The interviews were designed to elicit store managers’ and owners’ views about healthy foods, the local food environment, and the challenges and opportunities they face in creating access to healthy foods. We elicited perceptions of what constitutes “healthy foods” using two free-list questions. The study was designed and implemented in accord with principles of community-based participatory research.

**Results:**

Store owners’ and managers’ conceptions of “healthy foods” overlapped with public health messages, but (a) agreement about which foods are healthy was not widespread and (b) some retailers perceived processed foods such as snack bars and sugar-sweetened juice drinks as healthy. In semistructured interviews, store owners and managers linked the consumer food environment to factors across multiple levels of analysis, including: business practices such as the priority of making sales and the delocalization of decision-making, macroeconomic factors such as poverty and the cost of healthier foods, individual and family-level factors related to parenting and time constraints, and community-level factors such as crime and decline of social cohesion.

**Conclusions:**

Our results link food stores to multilevel, ecological models of the food environment. Efforts to reshape the consumer food environment require attention to factors across multiple levels of analysis, including local conceptions of “healthy foods”, the business priority of making sales, and policies and practices that favor the delocalization of decision making and constrain access to healthful foods.

## Background

In May 2010, the White House Task Force on Childhood Obesity identified food environments as a driver of childhood obesity and recommended a $400 million initiative to increase access to healthy foods in low-income communities in the United States [1]. This initiative and related efforts draw on evidence that neighborhood characteristics such as poverty and racial composition are associated with inequalities in access to food stores [2–8] and in the risk of obesity [9, 10]. The assumption is that unequal access to food stores constrains dietary choice and contributes to social inequalities in the risk of obesity and diet-related disease [11].

Research in this area has been guided by the distinction between community and consumer food environments [12]. The *community food environment* refers to the spatial distribution of food sources, including the location, density, and type of food stores. Evidence linking community food environments to health, so far, is mixed [13–15]. Some studies report associations with dietary intake [16–18], but others show no association [19–22] or associations in an unexpected direction [23, 24]. The inconsistency of results is partly attributable to differences in measurement [25], but it also likely reflects the distal relationship between community food environments and dietary behavior: The effect of community food environments on dietary intake depends on many intervening factors at the local level, including the quality of food stores and products available in them, local marketing and promotion strategies, the quality of service in stores, and residents’ perceptions of local food stores [26].

Research on the consumer food environment addresses this limitation. The *consumer food environment* refers to what consumers encounter in and around food outlets, including the placement, cost, quality, and promotion of specific foods. Previous observational research suggests that consumer food environments are linked to dietary intake in multiple settings. In a study of 12 communities in California and Hawaii, for example, the availability of low-fat and high-fiber foods in local retail outlets was associated with reported healthfulness of individual diets [27]. Among African American women in eastside Detroit, more positive perceptions of the selection and quality of fresh produce at local retail outlets was associated with higher fruit and vegetable intake, independent of store type and location and of individual-level characteristics such as income and education [28]. And in New Orleans, vegetable intake of local residents was positively associated with the amount of shelf space allotted to vegetables in food stores located within a city block of one’s residence [29].

These findings have led some researchers to target food stores in obesity prevention interventions [30]. A recent systematic review identified 16 interventions in small food stores over the 20 year period of 1990–2010 [31]. A common goal across all the trials was to increase the stocking of healthy foods, especially produce, low-fat dairy, and whole-grain breads; some interventions also tried to reduce the availability of unhealthy foods. Other common strategies included in-store signage to promote healthy foods, cooking demonstrations or taste tests to increase familiarity with healthy foods, stakeholder workshops to develop or refine interventions, and community meetings designed to engage store owners and community members. Results indicate that store-based interventions have modest but positive impacts on the availability, sales, and consumer knowledge of healthy foods [31].

Despite such promising results, however, we know relatively little about store owners’ and managers’ perspectives and experiences of factors that impact the consumer food environment and may affect the efficacy or sustainability of future interventions. Of the 16 trials identified by Gittelsohn and colleagues [31], for example, only three included interviews with food store retailers. Two of these studies [32, 33] reported process evaluations to assess store employees’ experiences of the interventions; one [34] reported formative research that led to a successful corner-store intervention. In all three studies, store owners were generally willing to participate in store-based interventions and helped identify potential barriers to increasing sales of healthy foods, including consumer demand and store infrastructure (e.g., refrigeration). Yet it is unclear whether lessons learned in these studies sufficiently inform store-based interventions in other settings.

This paper extends the evidence base for future food-store interventions by examining the perspectives of store owners and managers in Tallahassee, Florida. We conducted an exploratory, mixed-methods study to understand the business practices and contextual factors that influence decisions about what and how to sell the products available in food stores in Tallahassee. We also elicited store owners’ and managers’ perceptions of the food environment, including their expectations for how, if at all, they could increase access to healthy foods. In this sense, our study contributes to understanding how the consumer food environment comes to be and how best to intervene. We also argue that our findings enhance multilevel, ecological models of the food environment and challenge us to confront broader social structures that drive social inequalities in health.

## Method

### Research setting

Tallahassee is a small city in the panhandle region of Florida (USA), with an estimated population of 181,376. In the 2010 US Census, 57.4% of the population self-identified as White, 35.0% as Black, and 3.7% as Asian. Approximately 6% self-identified as Hispanic or Latino. As the state capital and home to two major universities, Tallahassee’s largest employment sectors include education, public administration, health care, and the arts and recreation. During 2006–2010, an estimated 28.5% of residents were living below the federal poverty level.

Previous researchers have documented spatial inequalities in the community and consumer food environments of Tallahassee and broader Leon County. Rigby et al. [35] examined the distribution of food stores in Leon County by neighborhood characteristics, including poverty and racial composition. They found that small grocery stores accepting Supplemental Nutritional Assistance Program (SNAP) benefits were more common in predominantly Black neighborhoods, but these neighborhoods had no supermarkets. Leone et al. [36] showed that store type and neighborhood characteristics are associated with the availability of healthy foods within stores in Leon County. In particular, (a) supermarkets offered the lowest prices for fresh fruits and vegetables, low-fat milk, and whole-wheat bread, and (b) stores in high-income neighborhoods devoted more shelf space to healthy foods than did stores in low-income neighborhoods.

**Figure 1:**
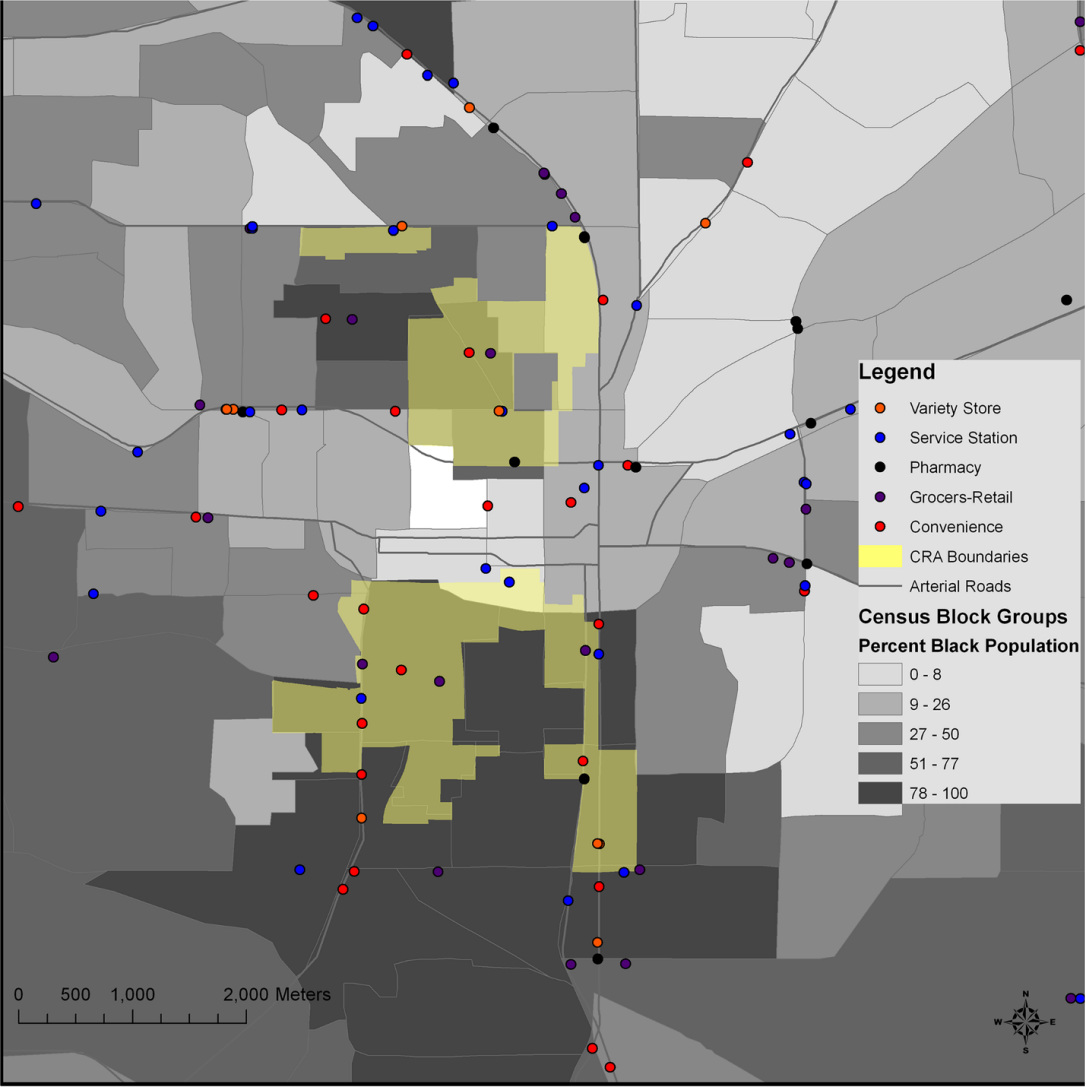
**Racial composition and the location of food stores in the vicinity of the Community Redevelopment Area (CRA), Tallahassee, FL, 2010.**

We explored the sociocultural processes driving these inequalities by eliciting the perspectives of food store owners and managers in the areas of highest need. Our work emerged from a community-academic partnership known as the Health Equity Alliance of Tallahassee (HEAT), which is grounded in principles of community-based participatory research [37]. Community partners and local policy makers directed our focus to two areas defined by the City of Tallahassee’s Community Redevelopment Agency (CRA) in 1998. The CRA includes two discrete redevelopment areas that border downtown: Greater Frenchtown and Southside (Figure 1). Both areas include predominantly African American neighborhoods with relatively high levels of poverty but also rich histories of identity, community, and civil rights activism [38, 39].

### Participants

The Leon County Health Department provided a complete list of 59 food stores in the vicinity of the CRA areas. We consulted with community-based organizations and local residents to ensure that the list was accurate and complete. From this list, we selected a purposive sample of 25 food stores to represent variation in store type and neighborhood characteristics (poverty and racial composition). Sample size was based on theory [40] and empirical research [41] about the minimum sample needed to capture variation in cultural knowledge and experience in a particular domain. The sample included two supermarkets, five grocery stores, eight convenience stores, four gasoline stations, and one pharmacy. We recruited one manager (n = 16) or owner (n = 4) from each store that agreed to participate. Potential participants in five stores refused to participate. Some managers indicated that corporate policy prohibited them from participating in research; others may have faced time pressures or may have doubted our explanation of why we were doing the study.

### Interview design

We conducted in-store interviews during business hours. This decision allowed interviewers informally to observe the context and flow of business in the store, but it increased the need to conduct interviews efficiently. We chose an open-ended, semistructured interview format to balance the need for efficiency with our exploratory goals of allowing participants to define the pace, content, and flow of the interview [42].

We drew on the local expertise of community partners and the experience of other communities and researchers in designing food-store interviews. The purpose was to give store owners and managers an opportunity to teach us about their perspectives and experiences regarding their business practices and the food environment. Questions were open-ended, and interviewers were encouraged to follow new leads introduced by participants. We included a free-list question [43, 44] in which participants were asked to list as many “healthy foods” as they could. The purpose of this technique was to elicit store participants’ perceptions of what constitutes healthy eating, so that subsequent intervention messages could be crafted in terms that are meaningful to store owners and managers.

The interview guide reflects input from university researchers (CCG), community partners (MMM, CB), and local high school and university students who conducted interviews in collaboration with the project coordinator (PQB) as part of a youth leadership development program. The University of Florida Institutional Review Board approved the study protocol (#2009-U-1148); we obtained written informed consent from all participants. Interviews were completed between February and June 2010.

### Analysis

Interviewers audio recorded 14 of the 20 interviews, and we made verbatim transcripts following standardized guidelines [45]. For six participants who did not consent to audio recording, we relied on notes that interviewers took during the interview and expanded immediately afterwards. We used the notes for context and interpretation; the data presented here come from verbatim transcripts of audio-recorded interviews.

We imported free-list data into ANTRHOPAC software (Analytic Technologies, Lexington, KY, USA) to calculate the frequency, average rank, and salience of the “healthy foods” that participants listed [46]. These results help to identify types of foods that store owners and managers consider to be healthy.

We imported interview transcripts and notes into MAXQDA 11 software (VERBI GmbH, Berlin, Germany), which facilitated both word- and code-based approaches to the analysis [47]. First, we examined word frequencies for the whole sample and separately by store type to identify potential themes that we later examined through close reading of the text [48]. Second, we read the texts to identify themes, or underlying dimensions of meaning, with a focus on repetition, similarities and differences within and between texts, and linguistic connectors (e.g., “because”, “then”, laughter) [49]. We identified themes inductively, based on patterns in the data, in keeping with our exploratory aims. The first author (CCG) developed a codebook to standardize definitions of themes and specify the conditions under which segments of text should be coded as instances of a theme [50]. He did the primary coding, with other team members contributing to interpretation and synthesis.

## Results

### Free lists of healthy foods

Respondents listed 96 items as “healthy foods” (mean number of items per respondent = 8.3, SD = 6.9, range = 2–33). Most items (77%) were listed by only one or a few respondents, as is common in free lists. Table 1 shows the 21 items that were listed by more than one respondent. Three patterns are evident.

**Table 1:** **Items listed by more than one respondent in free list of “healthy foods” (N = 19)**

	Item	Percentage	Average rank	Smith’s S
1	Vegetables	47	2.7	0.383
2	Fruit	42	4.0	0.274
3	Chicken	32	6.5	0.183
4	Cheese	32	7.0	0.153
5	Juice	26	3.8	0.160
6	Milk	26	4.0	0.182
7	Bread	26	6.4	0.126
8	Meat	21	4.5	0.077
9	Cereal	21	5.0	0.118
10	Fish	21	4.8	0.136
11	Peas	16	12.7	0.080
12	Nuts	16	6.0	0.094
13	Eggs	16	4.7	0.097
14	Soup	16	7.0	0.077
15	Salad	16	5.3	0.103
16	Baked foods	11	3.5	0.071
17	Greens	11	4.0	0.058
18	Oranges	11	12.0	0.034
19	Onions	11	5.0	0.046
20	Apples	11	11.0	0.047
21	Water	11	2.0	0.084

First, no item was listed by half of the respondents or more. *Vegetables* and *fruit* stand out as the most frequently listed items, but they were mentioned by only 47 and 42 percent of respondents, respectively. All other items were listed by fewer than one-third of respondents. Typically, this pattern indicates low agreement about what belongs in a cultural domain.

Second, in general, food-store managers and owners listed items that public health nutritionists would also identify as healthy foods. Fresh produce dominates the list, both in the generic categories *vegetables* and *fruit* and in specific items such as *peas, salad, greens, oranges, onions,* and *apples.* The list also includes dairy, low-fat meats (e.g., *chicken* and *fish*), and healthy cooking methods (e.g., baked foods). This pattern suggests opportunities to craft intervention messages using concepts and categories that are meaningful to store owners and managers.

Third, free-lists results raise questions about possible cultural differences between what store owners perceive to be healthy foods and what consumers prefer to eat. There is little evidence, for example, that store owners and managers’ perception of healthy foods is tailored to the cultural preferences of customers in the predominantly low-income, African American neighborhoods the stores serve.

Following the free-list question, we asked respondents to list the healthy foods, if any, sold in their store. Table 2 shows the 16 foods identified by more than one respondent. Again, the low frequency of all items indicates relatively little agreement among respondents. On average, respondents identified about half as many healthy foods in the store as they did in the free-list (mean number of items per respondent = 4.3, SD = 2.2, range = 1–8).

**Table 2:** **Items identified by ≥2 store managers and owners as “healthy items” offered in the store (N = 20)**

	Item	Percentage	Average rank	Smith’s S
1	Juice	35	3.4	0.188
2	Milk	35	3.0	0.213
3	Canned Vegetables	20	2.5	0.156
4	Cereal	15	4.0	0.088
5	Bananas	15	4.7	0.051
6	Orange Juice	15	2.7	0.087
7	Fruit	15	3.3	0.087
8	Chicken	10	1.5	0.093
9	Fruit Cups	10	1.5	0.087
10	Grapefruit Juice	10	4.5	0.051
11	Cereal Bars	10	1.5	0.093
12	Cheese	10	4.0	0.049
13	Apples	10	5.5	0.029
14	Granola Bars	10	1.0	0.100
15	Nutri-Grain Bars	10	3.0	0.073
16	Soup	10	4.5	0.044

In particular, respondents reported that few healthy foods, as defined by their free lists, are available in the stores. Vegetables and fruits dominate the free lists but are not commonly among the “healthy foods” available in the sampled stores. *Bananas* and *apples* were mentioned by multiple respondents, but no fresh vegetables were. Instead, processed foods are prominent in responses about the healthy foods available in stores. Such “healthy foods” include *canned vegetables, fruit cups, juice drinks, cereal*, and a variety of snacks. Perishable items are virtually absent from the list.

According to store managers and owners, the most common “healthy food” in stores is snack bars. The terminology for this category is not uniform but includes *cereal bars, granola bars, Nutri-Grain bars, Power Bars, health bars, energy bars,* and *protein bars.* If these items were collapsed into a single category, it would be the most common item of all (with a frequency of 10, or 50 percent).

### Thematic analysis

**Figure 2:**
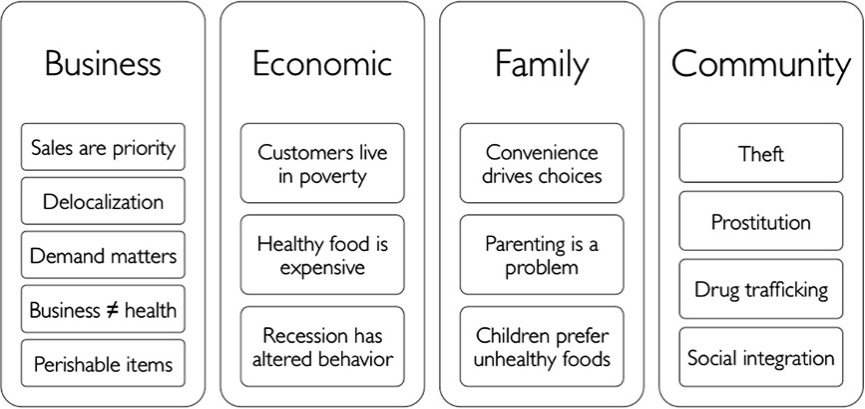
**Key themes in semistructured interviews with food-store owners and managers (N = 20).**

We have organized the themes from semistructured interviews into four levels of analysis: (1) store and business factors, (2) economic factors, (3) individual and family-level factors, and (4) community factors. Figure 2 summarizes the most salient themes in each area.

#### Store and business factors

##### Business priority is making sales

Not surprisingly, the top priority for managers and owners in all store types is to make sales. In the words of one drug store manager, “Sales is what drives it”. A local grocery store manager and the manager of a chain convenience store, respectively, offer typical explanations for how they decide what to sell:

Pretty much how fast it sells. Costs, definitely—cost plays a part of it. But…it’s pretty much how fast it sells, you know. We’re not gonna get it if it don’t sell. (SS14)^a^

You know, what they’ll [corporate purchasing] do, they’ll take a wide range of products. Say, for instance, food. We may get different types of food in here for six months. If that product turns a lot, then it’s gonna become a permanent product. If it doesn’t turn—we don’t get a whole lot of turns on it—then we’re not gonna continue to carry it. We’ll clearance it out. (QB02)

Another manager emphasized that the store sells “whatever the clientele wants. That’s from corporate down, ‘cause we’re sales-driven, obviously” (QB04).

##### Delocalization of decision making

The previous respondent’s reference to decision-making “from corporate down” highlights the delocalization of decision-making about which products to sell. Local managers generally perceive that they have little influence over the foods available in their stores, referring instead to “corporate”, “my corporate manager”, or “that’s a corporate thing”.

That’s really a corporate thing. That comes down from corporate, and district has some things—our regional and district office has, obviously, some say in it. But the majority of things come from our corporate office, ‘cause that’s where our purchasing department is at—our purchasing and marketing department. So, they kind of make a group decision, and they try to pass it along to all the stores, let’s say, in Florida. And so most of the stores in Florida are all gonna carry the same thing. It helps with distribution and pricing and things like that. (MR21)

The manager of a chain supermarket offered a similar explanation:

A lot of that comes from corporate. We can, if a customer requests something, we can go through corporate and request, “Hey, we’ve got a customer…” Late last week [I had a customer] wanting rice drink, the rice milk? And I’m trying to get that for her right now. It’s not approved at corporate, but I’m trying to get it for her. (QB04)

Corporate decision-making limits store managers’ perceptions of how they could influence access to healthy foods. The manager of a convenience store said: “As the store itself, we have no control over what we sell. So there’s nothing I can do on my part to make those changes” (QB06). A drug store manager elaborated:

Really, as an assistant manager, we don’t really have much say in what we carry. Yeah, we can put in our two cents, but even at the store manager’s level, unfortunately, it still goes back up to corporate. So, yeah, we can tell them, “We need this and we need that,” but, unfortunately, they’re gonna go off of what’s gonna be the best price, what they’re gonna be able to keep on the shelf the longest, because they can’t keep a lot of fresh foods. It would be nice to see some fresh fruits up at the front register and things like that, ‘cause I think they would sell…but I just don’t see us, at the store level, having enough influence to make those changes. (MR21)

The regional or national market of chain stores limits the ability to focus on specific communities. As one manager explained, “Companies like this that got 5,000 stores will—maybe only a few hundred might be in a neighborhood like this, so they try to offer a variety of stuff based on where you’re located, as a whole”. He continued:

Only 20 percent of the country is, is minority, or black, whereas the majority of the country is white, so you’re gonna carry more products that’s gonna cater to a certain population group than, you know… We don’t carry anything that would cater to a Hispanic, say… But in central Florida and south Florida they carry more products based on, you know, food products and hair care products based on that demographic…. So if we can get the companies to just concentrate more on the community itself, rather than as a whole, like a city or country—concentrate more on what the community needs…. (QB02)

The delocalization of decision making is clearest for stores that belong to regional or national chains; owners of independent grocery and convenience stores make their own decisions about what to sell in the store. Even independent stores, however, are constrained by non-local, corporate decisions made by the vendors they rely on:

So, [customers] ask me, “Well, you don’t have this one. Could we get that?” I say, “Yeah.” … So we’re open to suggestion. And that’s what I think, a business works best that way, too. But my vendors don’t believe it! They want to do what their corporation tells them. I say, well, I’m in between you and the customer. (QB01)

##### Consumer demand matters

One way store managers can try to influence corporate decision-making is by relaying customer feedback.

About the only thing I can really do is just continue to try to make, you know, give input back, feedback to the company, you know, through emails or what customers are interested in and may want to see in the store. And we try to do that on a weekly basis. (QB02)

This excerpt points to the importance of consumer demand as it relates to the priority of making sales. The perception of customer demand is most important in independent stores, where owners make their own decisions, but even managers of chain stores express this theme.

Most of the time, if some customer comes in and say that, uh, “You don’t have this product”, and they would like to have it, we go ahead and buy it. And if we see, if we sell more of it, we keep ordering it. If we don’t sell it, then, we buy the one time and next time, when the same customer wants it or somebody else wants it, that’s when we order it. (MR22)

Interviewer: What would it take for corporate to want to offer healthier foods in the store?

Demand. Public demand. Customer demands. And the popularity of how it’s selling in other areas and stuff. (QB06)

Actually, sometimes you have people that come in, and if they don’t see it in the store, they will say, like, “Why y’all don’t sell this?” or, “Why you don’t sell that?” or “Maybe you should try…. So, what I say to him [the owner]—because they’re foreigners—I would say, “OK, this is what our people like”, you know? And they get it! (SS15)

Some respondents identified lack of demand for fresh produce as a barrier to offering healthier food. This perspective was especially relevant for non-grocery stores, as the manager of a convenience store explained:

I guess we could probably carry a lot more perishable items as far as fruits and stuff like that, but, like I said, if they don’t pretty much come in and ask for it, we don’t carry it. And a lot of people are finicky about where they buy their products, you know what I mean? So, depending on the setup of your store and exactly where you’re at, you’re not just gonna come in and order, I mean, go grocery shopping in this store particularly. I don’t think anyone would come in and actually grocery shop. Given the area we’re in, I just don’t think they’re gonna go in and purchase fruits and stuff out of this place here. I just don’t think they would. (SS14)

##### What’s good for business is not necessarily good for health

The priority of making sales may clash with public health goals of increasing the availability and consumption of healthy foods. This conflict is evident in some respondents’ perception that what is good for business is not necessarily good for health.

The most item I sell, you know, contains a lot of sugar, like the Little Debbies, Honey Buns, which is not good these days because the kids don’t get enough exercise and don’t get involved in sports and stuff at all. So those are the food that’s not good for them. Sometimes they can eat donuts—but not every day. And I’ve seen some people buying it every day, sometimes two times a day, which is not good. And you know, the good thing is, you know, the good thing is the business. I do business because of that.

Interviewer: Right. It’s good for your business.

But, I mean, it’s not good for health. (SS18)

One respondent expressed dismay that processed snack foods—“the worst thing I ever, ever, ever”—were “the best sellers” in his convenience store (QB01). Another manager replied with embarrassed laughter when asked what he thought were some of the good things about the choices people who come into his store make: “Some of the good things? They’re high profit items for me” (QB06).

##### Stocking and selling perishable items is a challenge

One of the most commonly identified barriers to offering healthy foods is selling perishable items before they expire. This is a complex issue that involves low perceived customer demand, high risk of taking a loss on unsold items, and inadequate infrastructure for stocking perishable goods.

To begin, store managers and owners express an understanding that the healthiest food choices are perishable items. When asked about healthy foods in the store, a drug store manager replied:

We have some in the cooler. Most of our healthy—like I would consider real healthy stuff—is in the cooler. We have some, like, fruit cups. We’re just now carrying a grapefruit cup for in the morning, for breakfast alternatives. We don’t carry any fresh veggies or fruits. We used to, but the fruits—we mainly had fruits—but they were kind of going bad, so they had to take those out. (MR21)

Other respondents picked up on the challenge of selling items before they expire:

In another way, I’ve tried, you know, several other foods that are like, you know, lite. They call it ‘lite’. You know, snacks and stuff like that. So I tried to keep that in the store, but it went out of date, so I had to stop from keeping it. (SS18)

The short shelf life of perishable items is particularly challenging for owners of small, independent stores, who risk loss if items do not sell. One owner linked his perception of risk to low demand, expiration dates, and distributors’ policy of not accepting returns for expired items:

If I buy it, and it doesn’t sell, I have to throw it out. The milk guy doesn’t deliver anymore. I buy, myself, all the milk. If not, I have to throw it out or drink it myself. They [distributor] add to my minimums about a hundred, two hundred dollars worth of milk, and there’s no return on them either. … It’s demand. The supply is there, but then the supply is time-limited. It’s no return. That’s what the problem is. I cannot keep all the food they [customers] want because all the healthy food has dates on them.

Interviewer: OK, so the expiration dates are a problem?

Yeah, and the vendor doesn’t take it back. For me, you know, losses ought to be shared. Like a profit, we’re sharing. Why can’t we share the losses too? But they don’t. (QB01)

Many stores simply lack the infrastructure to stock perishable items. When asked what it would take to stock more healthy foods, the owner of a small convenience store said:

We’d probably have to get another refrigerator. Definitely a cooler or something to carry the meats in—I mean, given that we’re limited in space inside this place. We would definitely have to find some kind of way to fit a cooler into where we can actually carry, I guess, some fruits and maybe organic foods and whatnot. (SS14)

The emphasis on space and refrigeration was especially common in non-grocery stores. A gas station manager was clear about what it would take to stock more healthy foods: “I guess more space to put it. I got a smaller type of store here” (SS06).

#### Economic factors

##### Poverty constrains consumer food environment and healthy eating

Store managers and owners see that cost drives down demand for healthier foods. This problem was magnified during the recession of 2008–2010, but even in more prosperous times, respondents perceived that poverty shapes the types of foods people buy and how frequently they shop:

It’s a poor community, so a lot of people are basically eating what they can afford, which is like noodles, Ramen noodles and stuff like that—some canned goods. First of the month, you’ll have a customer maybe come in here and buy $83 worth of canned goods and noodles and juices because, basically, that’s what they can afford. You know, the lower, the cheaper end products (QB02).

Some stores extend credit to customers who do not have enough money to purchase food. This practice puts stores at risk, if customers default on credit payments:

Some people around here, when they come in to buy something, they don’t have enough, so we have to work around that and just tell them to bring it next time or something like that. We do have a credit account for some, a few people, so if they don’t have money right away, we can put it in the book for them, and when they have money, they come over here and pay it, so… Sometimes we forget, sometimes they forget, so that’s mostly the problem we have right now (MR22).

##### Healthy food is expensive

The economic challenges are compounded by the perception that healthier foods are expensive. The higher cost of healthy food has two consequences for food stores: (1) owners or purchasing managers make decisions about what to buy based in part on costs to the store; (2) the perceived higher cost of healthier foods lowers demand and makes it more difficult for stores to sell perishable items before their shelf life expires. A small grocery store owner summarized how these issues intersect, when asked about how he decides what to stock:

Can people afford it, first of all—affordability. And is it going to sell. That’s mainly the thing. If it does not sell, then I have to think twice. Mainly when I buy stuff for the store, I consider myself as a customer—if I’m going to buy it or not. Then I decide that, yeah, I can afford to buy it, then I know that my customers can afford to buy it. I put myself in their shoes. (QB01)

The manager of a small grocery store contrasted the costs of healthy and unhealthy foods in the store:

See, most everything right now is costlier. Beer is the only thing that’s the cheapest. We do have some juice that’s $1.99, and a lot of college students around FAMU, they buy them a lot. Well, if it were up to me, I would make sure that the price of food is cheaper than soda and everything. The cheapest thing we have is sweets and candy, and people, sometimes they have five or ten cents, they go ahead and buy a candy instead of saving that and collecting like a dollar or so, so they can buy some nice meat or some juice or something like that. (MR22)

Other store owners and managers emphasized that the costs of food can be reckoned both in terms of money and time:

It’s cheap. I mean, look at the stuff. You can go and buy a dollar hamburger, and you can’t go buy a dollar steak. Or a dollar piece of chicken and then take the time to cook it yourself. So, I think the convenience, and the culture behind that…. (MR21)

##### Recession changed consumer behavior

Store owners and managers reported that the cost of food became a more significant problem during the recession of 2008–2010. The recession was especially difficult for small, independent stores, where respondents lamented that “money is tighter” (QB01) and “it’s tough right now, very tough” (SS05).

It’s day-to-day getting so hard, and the economy doesn’t help at all. People used to buy $0.99 bag of chips. Now they’re looking for $0.25 bag of chips. They don’t buy that much. I had to look for alternates, as well. And so many of my vendors, they stopped coming here, because I don’t sell much. So, I had to look for alternates who can supply the same kind of stuff, so I had to go and shop around, too. And everybody’s got just the same price, you know, high price. (QB01)

Some respondents thought the recession reversed a positive trend of buying more healthy foods. A supermarket manager explained:

From my point of view, the past couple of years, since the economy has gotten to the point it’s at, it has actually regressed some—people aren’t spending as much money, trying to save money where they can. But prior to that, we were seeing an increase in more organics, more fat-frees, more whole wheats, etc. (QB04)

#### Individual and family factors

Store managers and owners also identified individual and family-level factors as determinants of unhealthy behaviors. Some respondents linked these factors to structural constraints (e.g., poverty, household composition), while others emphasized what they perceived as cultural patterns.

##### Convenience drives food choices

Many respondents perceived that convenience drives food choices. This perception relates to the earlier theme of cost: If people lack money or time to prepare food on their own, they may resort to processed or prepared foods:

Yeah, definitely convenience. I mean, versus you going into the kitchen and preparing a meal, that would save you, what? Taking about thirty minutes to prepare a meal versus you just going to McDonald’s and getting it within five minutes. I mean it’s definitely convenience. … It’s just a lot easier to go and pick it up versus you going in and preparing it yourself. (SS14)

While some respondents regard time poverty as a factor, others invoked cultural explanations, citing the decline of cooking, the pace of lfe, or a desire (promoted by advertising) for convenience.

Looks like they need frozen, ready food, you know? Yeah, put it in a microwave, finish it up. Lately, the young generation don’t like to cook, no. They want to go prepared. (SS05)

Probably the culture we’re in is too quick, fast. We’re gonna go after fast food before we’re gonna go home and cook us a meal, or warm us up some soup. (MR21)

It’s quick, convenient, and it’s advertised, and that’s what they have on their mind that they want. (QB06)

##### Parenting is a problem

Some store owners and managers see parenting as a source of unhealthy food choices. One respondent labeled the problem as “lazy parents”. A more common explanation was that parents are under time or economic pressure and do not have time to cook for themselves or to teach children about nutrition. Whatever the cause, respondents commonly linked children’s food preferences and purchasing decisions to values they learned at home:

Because they have—I can’t say ‘no home training’. Their parents don’t push them to eat healthy stuff in the house. I betcha no house has apples, bananas, and oranges, like mine. My baby, I push him to eat stuff like that, even though he say he don’t want it. If that’s all you got, what you gonna eat as a snack, you know? He say, “Ma, go buy Little Debbie”. No, I buy apples, oranges…” (PS25)

##### Children prefer unhealthy foods

Whereas some respondents attributed children’s preference for junk food to poor parenting, others saw it as an inevitable part of childhood. When asked why children buy junk food, for example, one convenience store manager replied, “It’s just kids being kids, you know?” (QB02). A supermarket manager agreed and related this observation to his own (presumably well parented) children. In reply to the question of why children buy junk food, he said:

As far as why they pick ‘em? Because they’re not healthy. [laughter] I’ve got two little girls. They wanna eat junk. [laughter] Like the Capri-Sun? That’s a big thing. And the Kool-Aid Jammers—among the kids, we sell a lot of these. (QB04)

Not all respondents explained why they thought children would prefer unhealthy food over healthier options, but one grocery store manager suggested that it could be an act of rebellion: “I guess they wanna be bad”. But then he noted: “Although, when they’re good, they get it as a reward. [laughter]. I don’t know” (SS16).

As a component of consumer demand, children’s food preferences relate to the core priority of making sales. Thus, as one grocery store manager acknowledged with uncomfortable laughter, children’s food preferences influence marketing decisions:

Definitely the snacks. I would say the hard candy, the chocolates, ice cream, the chips. Anything that’s not healthy, that’s what they come here for. [laughter]

Interviewer: So why do you think they buy those foods?

I think that’s just something that started early, at an early age, where they’re giving the kids sweets and whatever. They get used to it. It’s almost as a treat, you know what I mean? You almost rewardin’ ‘em with something, so they get used to it, so that’s what they’re gonna come in and ask for. And that’s pretty much all they come in and get. That’s mainly why we carry what we carry. (SS14)

#### Community factors

Store owners and managers identified community-level factors as among the most important challenges facing their business. These factors include theft, loitering, drug trafficking, prostitution, and other criminal behavior near the store. Some respondents also identified a lack of community cohesion as a challenge to their business.

One supermarket store manager said the biggest challenge is “keeping everything at the front cleaned up” (QB06). Other respondents identified illicit activity such as theft, prostitution, and drug trafficking as key challenges:

Our biggest challenge is shoplifting and solicitation outside the store. It’s kind of an ongoing problem. You know, we’ve even had prostitution in the parking lot and just, you know, a lot of people out begging. So we get a lot of interaction with the customers, telling us that we got somebody out in the parking lot begging. So we deal with the police a lot, where we have to get them out here to kind of help us handle that, plus the shoplifting, so that’s our biggest issue. (QB02)

Dealing with these challenges increases the cost of running the business because it requires having more staff: “I have to make sure I have enough people on coverage to make sure that we can keep it clean, as well as customer service” (QB06). These costs are then passed on to consumers:

Interviewer: What are the biggest challenges you face in keeping your business going, and how to you deal with those challenges?

Theft. I mean, we put ‘no trespassing’ warnings, we send ‘em to jail, we try to raise prices to back up our inventory, when it falls short. That’s how we make up our inventory. (PS25)

Theft, prostitution, and drug trafficking pose a dual challenge to store owners and managers because such activities not only drive up costs but also may drive away customers. A manager of a small grocery store explained that the biggest challenges to her store are “outside activities”:

The young people hang around the store. No matter how much we call the police and ask them to pass out trespassing warnings, they still come back. Traffic is kind of congested, too, certain times of day, because of the activities outside.

Interviewer: So do you mean as far as—is it like juveniles hanging out or drug dealing?

It’s juveniles, grown people, drug dealers. Just, man, keeping it real, yes.

Interviewer: You think those prohibit certain people from shopping here?

Yes it does. It does. (SS15)

This store manager explained that she thinks the store has a role to play in organizing people in the neighborhood to reclaim ownership of the community. Here is her explanation of how to deal with the “outside activities”:

And with me asking other people to get involved in the community—let’s take our community back. I think that calling the police and other people getting involved is helping us out a little bit, too. … I’ve spoken to a lady named [name]. She says she’s a homeowner association president. And I’ve gotten her involved in this also, and we talk constantly about it. We’re supposed to be getting a meeting together because the homes in the community won’t sell. The property value is going down. And we just need to get—we, the people, need to get more involved. Even the church, the ministers over here need to get involved. (SS15)

As she continued, this store manager connected community redevelopment back to the consumer food environment:

You know, especially with my older customers. I have a lot of seniors, see, and that’s one of the main reasons, too, why I want the community to get involved. I want the senior citizens to be able to come back to their roots—you know what I’m saying?—without feeling threatened by the people that’s hanging out front and, you know, feeling uncomfortable with coming in and spending their money, thinking that they’re gonna get robbed. I’m not saying that those guys would do that…but, you know, some people just don’t have that feeling, that sense of feeling… (SS15)

## Discussion and conclusions

The purpose of this exploratory study was to understand the business practices and contextual factors that influence decisions about what and how to sell the products available in food stores in Tallahassee, FL. We presented systematic evidence regarding how food-store owners and managers conceptualize “healthy foods” and identified factors at multiple levels of analysis that shape their view of the food environment and their role in providing access to fresh, nutritious foods. The findings enhance multilevel, ecological models of the food environment and imply that reducing social inequalities in access to healthful foods will require interventions across the spectrum of prevention [51].

Our work contributes to a growing literature on the role of food stores in obesity prevention [31]. We identified six previous studies involving open-ended interviews with food-store owners [32, 52–56]. Our results confirm several key findings from this literature, including the priority of making sales, store owners’ perception of low customer demand for fresh produce, the challenge of selling perishable foods, and lack of space or infrastructure to stock fresh foods. Store-based interventions designed to address these barriers generally have shown modest, positive impacts on availability, sales, and consumption of fresh foods [31, 57], and at least one trial reported an impact on body mass index [58].

Our study builds on this work in three ways. First, we present systematic evidence regarding how store owners and managers conceptualize “healthy foods”. This contribution is significant because it could help to frame future store-based interventions in terms that are meaningful to food retailers. In particular, our findings suggest that store owners’ and managers’ preconceptions about healthy foods overlap with public health messages, but that (a) agreement about which foods are healthy is not widespread, (b) store owners’ perceptions of healthy foods may not be culturally tailored to communities they serve, and (c) processed foods, including sugar-sweetened beverages and snack bars, are among the “healthy” foods that owners and managers recognize in their stores. Future interventions will need to address these conceptions of healthy foods to be effective.

Second, our study highlights the need to address the financial implications of public health interventions for food-stores’ bottom line. The central theme in respondents’ explanations of their business is that, as one store manager put it, “sales is what drives it”. At first glance, the centrality of sales seems obvious. Bleich [59] argues, however, that public health researchers have not adequately addressed the priority of making sales. In particular, she suggests, existing food-store interventions (a) do not include sufficient fiscal measures to demonstrate short- and long-term impacts on profitability and (b) do not simultaneously consider the diverging goals of business owners and public health researchers [59]. Our results underscore the urgency of this critique. Store owners and managers in our sample articulated the primacy of making sales as the basis for all decision-making and acknowledged that what is good for business is not necessarily good for public health. Future interventions need to accommodate this perspective and provide better evidence regarding the fiscal impacts of store-based interventions. Without such evidence, it is unclear whether interventions are scalable, and it will be difficult to secure necessary buy-in from food-store owners.

Third, our findings extend multilevel, ecological models of the food environment. Story et al. [26] propose an ecological model of influences on what people eat, including individual-level factors, the social environment, the physical environment, and macro-level structures and policies. Our results suggest that the model applies not only to what people eat but also to what is available in food stores. Individual-level factors evident in store owners’ and managers’ interviews include perceived low consumer demand, perceived customer desire for convenience, parenting practices, and children’s food preferences. Social environmental factors include household structure, neighborhood crime, and community mobilization for economic development. The physical environment includes store managers’ and owners’ perceptions of neighborhood conditions and the lack of space or infrastructure to stock healthy foods. Macro-level factors include the delocalization of decision making, the primacy of commercial interests over public health concerns, food prices, poverty, and overall economic conditions.

One implication of this framework is that efforts to reshape the consumer food environment require interventions across the spectrum of prevention. Escaron et al. [57] observe that the most successful store-based interventions combine multiple strategies (e.g., point-of-purchase information, advertising and promotion, pricing) to increase both the supply of and demand for healthful foods. Our study, based on food-store owners‘ and managers’ understanding of their business, further suggests that store-based interventions would be enhanced by working systemically in other aspects of the food environment. For example, the delocalization of decision-making about what to sell in stores, which our study is the first to report, implies a need (a) to address corporate purchasing decisions and regional food-distribution chains and (b) to promote local sourcing of fresh foods [4]. Our respondents’ perception that healthful foods cost more—in time as well as money—resonates with evidence regarding the association between parental employment and childhood obesity [60, 61], with the multiple meanings of price among consumers [62], and with agricultural and pricing policies that produce abundant low-cost, energy dense, and nutrient poor foods [63–65].

Finally, the ecological framework also encourages us to look beyond the food environment for potential solutions. Our respondents made clear that some of the biggest challenges they face have little to do with food. Rather, they identified “outside activities” such as theft, prostitution, and drug trafficking as factors that increase their costs and drive away potential customers. This finding corroborates a report from Nashville, where food store owners perceive crime as the most significant barrier they face [56]. Our respondents also linked their business practices to broader conditions such as the decline of neighborhood social integration and concentrated poverty. Their insight reminds us that, in addition to individual- and community-level health promotion, we must also confront social structures that produce unequal access to power and resources necessary to achieve health [66, 67].

We acknowledge several limitations of study design. The small sample size, although appropriate given our exploratory aims, limits our ability to make systematic comparisons among store types, and we are unable to assess whether store owners and managers who agreed to participate differ systematically from those who refused. We also lack data to test whether owners’ and managers’ perspectives are associated with the availability of healthful foods, sales, or customer characteristics. Last, our focus on food stores in low-income neighborhoods identified by Tallahassee’s Community Redevelopment Agency (CRA) is justified by evidence of high need in the area [35, 36], but it limits our ability to generalize to other parts of the city or to explore how food-store owners‘ and managers’ perspectives may vary by neighborhood-level socioeconomic status.

Despite these limitations, this study contributes new insight into the business practices and sociocultural processes that shape local food environments. Future efforts to explain and eliminate social inequalities in access to healthful foods will be strengthened by incorporating a better understanding of store owners’ business priorities and perspectives, as well as the broader influences across multiple levels of analysis on the construction of consumer food environments.

### Endnote

^a^We include anonymous respondent identification codes (e.g., SS14) to make more transparent our use of the full data set in selecting illustrative excerpts.
